# Glioblastoma glycolytic signature predicts unfavorable prognosis, immunological heterogeneity, and ENO1 promotes microglia M2 polarization and cancer cell malignancy

**DOI:** 10.1038/s41417-022-00569-9

**Published:** 2022-12-09

**Authors:** Xisong Liang, Zeyu Wang, Ziyu Dai, Hao Zhang, Jian Zhang, Peng Luo, Zaoqu Liu, Zhixiong Liu, Kui Yang, Quan Cheng, Mingyu Zhang

**Affiliations:** 1 Department of Neurosurgery, Xiangya Hospital, Central South University, Changsha, 410008 P. R. China; 2 National Clinical Research Center for Geriatric Disorders, Changsha, 410008 P. R. China; 3 Department of Oncology, Zhujiang Hospital, Southern Medical University, Guangzhou, 510000 P. R. China; 4 Department of Interventional Radiology, The First Affiliated Hospital of Zhengzhou University, Zhengzhou, Henan China

**Keywords:** CNS cancer, Tumour immunology, Biomarkers, Cancer microenvironment

## Abstract

Glioblastomas are the most malignant brain tumors, whose progress was promoted by aberrate aerobic glycolysis. The immune environment was highly engaged in glioblastoma formation, while its interaction with aerobic glycolysis remained unclear. Herein, we build a 7-gene Glycolytic Score (GS) by Elastic Net in the training set and two independent validating sets. The GS predicted malignant features and poor survival with good performances. Immune functional analyses and Cibersort calculation identified depressed T cells, B cells, natural killer cells immunity, and high immunosuppressive cell infiltration in the high-GS group. Also, high expressions of the immune-escape genes were discovered. Subsequently, the single-cell analyses validated the glycolysis-related immunosuppression. The functional results manifested the high-GS neoplastic cells’ association with T cells, NK cells, and macrophage function regulation. The intercellular cross-talk showed strong associations between high-GS neoplastic cells and M2 macrophages/microglia in several immunological pathways. We finally confirmed that *ENO1*, the key gene of the GS, promoted M2 microglia polarization and glioblastoma cell malignant behaviors via immunofluorescence, clone formation, CCK8, and transwell rescue experiments. These results indicated the interactions between cancerous glycolysis and immunosuppression and glycolysis’ role in promoting glioblastoma progression. Conclusively, we built a robust model and discovered strong interaction between GS and immune, shedding light on prognosis management improvement and therapeutic strategies development for glioblastoma patients.

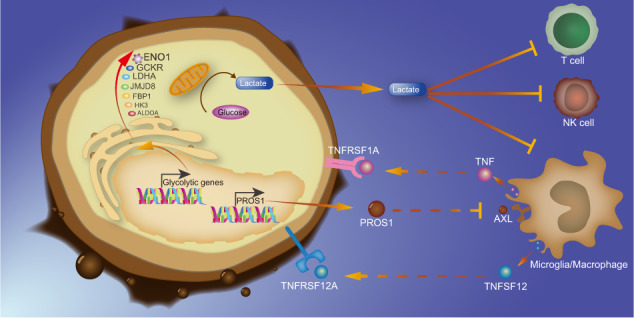

## Introduction

Glioblastomas (GBMs) are the most common and malignant tumors among brain tumors and lead to a high death rate [1]. The main lethal causes attribute to frequent recurrence and therapeutic resistance. Routine treatments of GBMs, including surgical resection, chemotherapy, and radiotherapy, did not reverse the poor prognosis essentially. Therefore, more effective approaches are urgently required. The prediction models offered novel methods that predict the risk probability of the clinical outcome. Previous models adopting anatomical, pathological, or clinical predictors showed limited predictive accuracy; the genetic panels have emerged as novel signatures that exhibited a more individualized property and precise predictive ability, and are being increasingly utilized as promising prognostic predictors [2, 3].

Aerobic glycolysis is an abnormal metabolic process adopted by tumor cells to obtain excessive energy and materials for their uncontrolled growth and metastasis [4], while the non-tumor cells also reprogrammed their metabolism to enhance aerobic glycolysis, switching to tumor-supporting cells [5]. In gliomas, the tumor cells utilize aerobic glycolysis not only to accelerate ATP acquisition but also to generate various metabolic production for tumor growth and proliferation, modulate the signaling network, and regulate the epigenetic network, which manifests that tumor glycolysis is a prognostic indicator and a novel promising therapeutic target [6].

The immune microenvironment contains massive components that play critical roles in glioma progress [7]. Their anti-cancer immunity suppresses tumor growth, but they can also be subverted by tumors and escape tumors from surveillance, for which immunotherapy has been a hotspot therapeutic approach [8, 9]. Currently, the interaction between glycolysis and the immune microenvironment has been found to interfere with tumor progress. For instance, glioma-associated macrophages (GAMs) can speed up the tumor glycolytic rate by phosphorylating glycolytic enzymes [10]. The non-tumors cells can also be affected to present immunosuppressive effects by activated glycolysis maintenance [11]. However, the critical nodes of glycolysis and their effects on prognosis and immune pathways or immunocytes are still sealed in gliomas.

To estimate the prognostic values of glycolytic signatures, Chen et al. [12] and Wang et al. [13] established a glycolytic model to predict survival, but their predictive performance was not estimated. Zhou et al. [14] built an energy metabolism-related signature risk score, including glycolysis. These genes were annotated with several immune-related biological processes. However, the independent correlations between glycolysis and immune were not presented, and no further analysis of these immune processes was conducted. To construct a robust, reliable model and elaborate the association between glycolysis and the immune, we conducted the Elastic Net algorithm to establish a Glycolytic Score (GS), validated it in large independent datasets, and conducted a series of analyses regarding immune pathways, immunocytes infiltration, and immune escape genes. Besides, the glycolytic genes and the GS were verified in a combined single-cell GBM dataset. The immune functions and intercellular cross-talk were compared between GS high and low neoplastic cells, and finally, immunofluorescence, clone formation, CCK8, and transwell assays were performed to confirm the roles of *ENO1*, the key gene of the GS, in affecting M2 microglia polarization and glioblastoma cell malignancy.

## Materials and methods

### Patient datasets and gene set

We collected 137 and 136 samples with sequence data from The Cancer Genome Atlas (TCGA) and Chinese Glioma Genome Atlas datasets (CGGA-seq), respectively, and 124 samples with microarray data from the CGGA dataset (CGGA-array) [15]; their basic information was listed in Table 1. TCGA dataset was used as the training set, and CGGA-seq, and CGGA-array datasets were used as validation sets. The single-cell RNA sequence data (GSE138794, GSE84465, scp50, and GSE131928) were downloaded from Single Cell Portal and Gene Expression Omnibus, processed according to the standard “Seurat” running procedure and the doublets were removed then. Glycolytic genes were retrieved from Gene Ontology (GO).

**Table 1:** The clinical information of the samples in three bulk datasets.

Characteristics	TCGA-seq (*n* = 137)	CGGA-seq (*n* = 136)	CGGA-array (*n* = 124)
*Gender*			
Female	47 (34.3%)	50 (36.8%)	48 (38.7%)
Male	90 (65.7%)	86 (63.2%)	76 (61.3%)
Age (mean (SD))	58.7 (13.5)	46.7 (12.6)	46.4 (12.3)
Age (median [MIN, MAX])	60 [21,85]	48 [8,79]	48 [12,70]
*Vital status*			
Alive	31 (22.6%)	13 (9.6%)	15 (12.1%)
Dead	105 (76.7%)	121 (89.0%)	108 (87.1%)
Censored	1 (0.7%)	2 (1.5%)	1 (0.8%)

This table presented the clinical characteristics of the samples in TCGA, CGGA-seq, and CGGA-arrya datasets.

### Prognostic clustering and GS construction

Consensus clustering and principal components analysis visualization (PCA) for TCGA samples was conducted based on the glycolytic genes. The prognostic prediction ability of clusters was examined by the Kaplan–Meier curve in training and validation sets. Heatmaps were plotted to present the glycolytic gene expression patterns and patient feature differences divided by the clusters.

The genes firmly associated with survival were filtered from glycolytic genes by the univariate analysis. The mutual correlations of the genes passing the univariate analysis were calculated, and the Elastic Net [16] was conducted with lambda.min to select the candidate predictors with coefficients. The GS was then calculated for each patient in training and validating datasets by the formula:

$${\rm{Glycolytic}\,{\rm{score}\,({\rm{GS}})}} = \mathop {\sum}\limits_i^n {\beta _i^ \ast\, g_i}$$

Where *g*_*i*_ means the expression of glycolytic gene *i*, *β*_*i*_ represents the coefficient of gene *i* retrieved from the Elastic Net.

### The estimation of the GS predictive ability for clinical and molecular features

We performed survival analysis to predict patient overall survival (OS) by the median GS value, which separated patients into the high-and the low-GS groups. The receiver operating characteristic curve (ROC) was used to estimate the prediction discrimination for OS, and IDH status in TCGA, CGGA-seq, and CGGA-array datasets.

We compared the GS levels between age groups, subtypes, IDH mutant/wildtype, 1p/19q codeleted/non-condeleted, and MGMT methylated/unmethylated. The cutoff value for age groups was set 45 years old. We subsequently compared the prognostic value of the GS with the previously established glycolysis-related model for glioblastoma [12, 13] by ROC and c-index. Furthermore, we presented the glycolytic gene levels with patient features as well as the GS in the training set. The expression levels of the 11 genes that passed the univariate analysis were also exhibited in the validating sets.

Single nucleotide variant (SNV) data were retrieved from TCGA, and the top 20 SNVs of two GS groups were presented by waterfall plots. Hypoxia signatures were collected from pan-cancer hypoxia studies [17–28], and the distribution of this signature was presented by heatmaps in training and validating sets.

### Functional analysis and immune cell infiltration

To investigate how the immune microenvironment is engaged in GBMs and affected by the glycolytic signature, we performed Gene set variation analysis (GSVA) [29], which converts the expression levels of single genes to biologically relevant gene sets, to seek the differences of Kyoto Encyclopedia of Genes and Genomes (KEGG) pathways [30] and GO biological processes (BP) variations in TCGA dataset, the immune-related terms were shown in the heatmaps. Gene Set Enrichment Analysis (GSEA) of KEGG [30] and GO pathways was applied to visualize the pathway enrichment score, which was calculated according to the whole gene expression ranks contained in each pathway.

Subsequently, the immune infiltration differences between the two GS groups were further compared by leukocyte signature matrix (LM22) [31] using the Cibersort algorithm [31] and 28 subpopulations of tumor-infiltrating lymphocytes (TILs) [32] calculation in both training and validating datasets. The diversity of the two immunocyte signatures between the GS groups was shown by the box plots, and the correlation between the immunocyte signatures and GS was presented by Lollipop charts.

### ESTIMATEScore and immune escape genes

Considering that the non-tumor environment consists of both immune cells and stromal cells, we then applied an Estimation of sTromal and Immune cells in mAlignant Tumor tissues using Expression data (ESTIMATE) [33] algorithm to calculate the Immune Score, Stromal Score, ESTIMATE Score, and tumor purity for each patient in TCGA, CGGA-seq, and CGGA-array datasets, and their level differences between the GS groups were exhibited by raincloud plots.

We also compared the expression differences of the immune escape-related genes between the low-GS and the high-GS groups in the TCGA dataset, the genes sets were obtained from a study by Wang et al. [34], on cell adhesion, antigen recognition, co-inhibit, co-stimulate, ligand, receptor, and other immune processes.

### The GS and 7 glycolytic gene expressions in a combined single-cell RNA-seq dataset

scRNA-data were processed using the R package ‘Seurat’ [35]. Data were normalized, and highly variable genes were selected, followed by data scaling, dimension reduction, and doublets removal. The single datasets were then integrated for PCA reduction, cellular cluster identification, and U-map reduction. After cells were clustered, the cells were annotated by corresponding markers, and the neoplastic cells were identified using the CopyKat algorithm [36], a tool to reckon malignant cells by calculating the aneuploid score. Then, GS and its componential seven glycolytic genes expression were exhibited in all annotated cell clusters.

### DEGs and Immune-related Functional analysis in the scRNA-seq dataset

We compared immune signature pattern similarity among all scRNA clusters by performing GSVA on GSEA immune signature. Subsequently, the neoplastic cells were divided into the low-GS and high-GS groups by median GS cutoff. The top 50 DEGs between the two groups were presented in heatmaps. GSVA [29] was conducted to seek the highly variated immune pathways from GO between the two groups. To clarify the enrichment levels of immune pathways with high diversity, we then conduct GSEA to obtain the quantified results. The corresponding normalized enrichment scores of immune terms were presented by the Lollipop chart, and the pathway gene positions in ordered gene ranks were exhibited by the GSEA plot.

### Intercellular cross-talk analysis for single-cell clusters

The intracellular cross-talk between all single-cell clusters was analyzed based on the ligand-receptor pair expression by the tool CellChat [37]. Dot plots were applied to present the outgoing communication patterns of secreting cells and the incoming communication patterns of targeting cells by the ‘netAnalysis_dot’ function. The intercellular communication networks were exhibited in circle plots by the ‘netVisual’ function, and the ‘netAnalysis_signalingRole_network’ function was used to show the role importance of the sender, receiver, mediator, and influencer.

### ENO1 gene interference, overexpression, and co-culture

The T98G cells and HMC3 cells were cultured in DMEM containing 10% fetal bovine serum and 1% penicillin/streptomycin. For siRNA knockdown, the T98G cells were transfected with ENO1-NC and ENO1 siRNA, (sequences listed in Supplementary Material S1) and transfection reagent for 48 h. For overexpression, ENO1/control lentivirus was added to the T98G cells with transfection reagent (40 µl HitransG A + 40 µl HitransG P, Genechem Co. Ltd., Shanghai, China). After transfection for 12 h, we replaced the transfection system with a fresh medium. Subsequently, T98G/si-ENO1-NC#, T98G/si-ENO1-1#, and T98G/si-ENO1-2 cells were co-cultured with HMC3 cells, the T98G cells, HMC3 cells were cultured in the upper and lower compartment, respectively. After 72 h, the cells were digested and collected for further analysis. The same methods were applied to T98G/Control, T98G/si-ENO1-1#, and T98G/si-ENO1-1-OE groups for the co-culture rescue experiment. The siRNA-knockdown and co-culture experiments were repeated independently three times.

### Western blotting detection of ENO1 protein expression

The T98G cells were washed with cold PBS and then lysed with RIPA lysis buffer. The mixture was further lysed by sonication. BCA quantification was conducted to quantify the concentration of proteins. The mixture of prepared total protein and loading buffer was separated by SDS-PAGE electrophoresis. After proteins were transferred to the membrane and blocked with 5%, skim milk, anti-ENO1 (Proteintech, US, 11204-1-AP), anti-β-ACTIN (Proteintech, US, 66009-1-Ig), and secondary antibodies incubation was sequentially performed (Goat anti-Mouse, AWS0001; Goat anti-Rabbit, AWS0002; Abiowell, China). Finally, ECL chemiluminescence was performed to visualize the bands. The experiments were repeated independently three times.

### Immunofluorescence assay of M2 macrophage markers

The HMC3 cells, after co-culture with T98G cells, were digested and seeded on the cell climbing slices. After washing with PBS, the slices were fixed with 4% paraformaldehyde and permeabilized with 0.3% Triton X-100. Then splices were blocked with BSA and incubated with anti-CD68 (ThermoFisher, US, 14-0688-82), anti-CD163 (Proteintech, US, 16646-1-AP) overnight and with fluorescent-labeled secondary antibodies for 90 min. The nucleus was stained with DAPI, and splices were sealed using glycerin. PBS washing was performed before each step above for 5 min, three times. The splices were observed under the fluorescent microscope. The experiments were repeated independently three times.

### Rescue experiment of glioblastoma cell clone formation, CCK8, and transwell migration assay

We digested the cells in different groups using 0.25% tryptase containing 0.02% EDTA to single cells and resuspended the cells in the medium for further assays. To estimate the cell clone formation ability, we seeded the cell with 500 cells per dish in a 2 ml medium and cultivated them in the incubator under 37 °C, 5% CO_2_. After 2 weeks, we removed the medium and washed the cells with PBS twice carefully. One milliliter of 4% paraformaldehyde was used to fix the cells for 15 min, and 1 ml crystal violet was added to stain the cells for 30 min after the paraformaldehyde was removed. Then, the cells were washed slowly and dried in the air, and the images were taken under the microscope. For the CCK8 assay, we seeded the cells per well in the 96-well plates with 5 replicates. After cells adhered to the bottom, the supernatant was replaced by 100 ml fresh medium containing 10% CCK8 reagent (DOJINDO, Japan, NU679) at 0, 24, 48, 72, and 96 h time points, and the OD value was detected by the microplate reader after cells cultivated with working fluid for 1 h.

Transwell cultivation was subsequently performed to evaluate the migration capability of the T98G cells. We resuspended the digested cells with medium containing no FBS and seeded 100 μl 2 × 10^6^/ml cells in the upper chamber, and added 500 μl complete media to the lower chamber. After 48 h cultivation of the transwell system, the upper chamber was washed with PBS three times, and the unmigrated cells were wiped off with cotton. We then fixed the cells using 4% paraformaldehyde for 20 min and subsequently stained them with 0.1% crystal violet for 5 min. Finally, the cells were washed and counted under a microscope. All experiments were repeated independently three times.

### Statistical analysis

All statistical analyses were performed using the R software. Mean ± SD was used to present the center values and the error bar in the figures. The Kaplan–Meier survival curves and log-rank test compare the differences in survival rates between the two groups. Student’s *t-*test and one-way ANOVA test were applied as appropriate to compare differences of normally distributed parameters between groups, and non-normally distributed parameters were tested by Wilcoxon test or Kruskal-Wallis test. Univariate analyses and elastic net regression were used to select the candidate and prognostic predictors. ***, **, *, NS represents *p* < 0.001, <0.01, <0.05, and not significant, respectively.

## Results

### Consensus clustering and construction of the GS

**Fig. 1:**
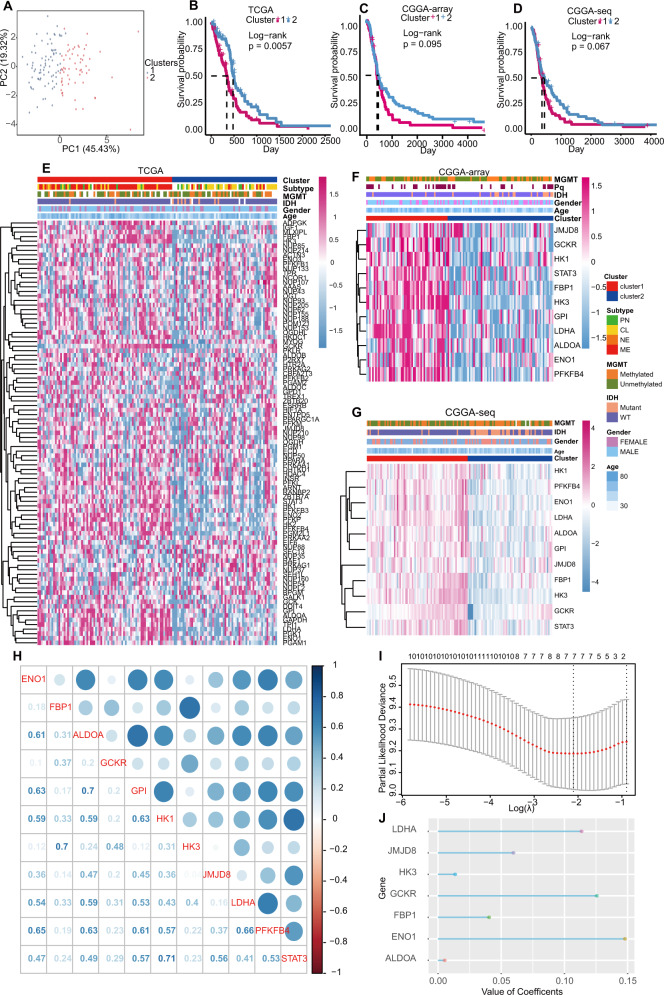
Consensus clustering and the GS construction by glycolytic genes. **A** A total of 137 patients from TCGA were divided into cluster 1 and cluster 2 by consensus clustering. **B**–**D** The Kaplan–Meier curves of clusters 1 and 2 separate the overall survival of patients from TCGA (**B**), CGGA-array (**C**), and CGGA-seq (**D**), respectively, into two prognostic clusters. **E**–**G** Heatmaps of differentially expressed genes ranked by clusters in TCGA (**E**), CGGA-array (**F**), and CGGA-seq (**G**), respectively. **H** Mutual correlations among 11 survival-associated glycolytic genes. **I** Candidate parameters identification by Elastic Net analysis, the final parameters were selected according to the partial likelihood of deviance. **J** The coefficients of selected seven glycolytic genes were calculated by the Elastic Net analysis.

The glycolytic genes were downloaded from GO via http://www.gsea-msigdb.org/gsea/msigdb/cards/. Two distinct clusters were obtained when consensus clustering was run based on these glycolytic genes in the TCGA dataset, they were defined as cluster 1 and cluster 2, as shown in Fig. 1A. To testify whether the clusters affected patient survivals, we plotted Kaplan–Meier curves in both training and validating datasets and found that patients in cluster 1 suffered from lower survival probability than those in cluster 2 (Fig. 1B) in TCGA. Though not significant, the same trend was also observed in CGGA-array and CGGA-seq datasets (Fig. 1C, D).

The glycolytic genes expression levels and clinical and molecular features were compared between cluster 1 and cluster 2 in the TCGA dataset (Fig. 1E); cluster 1 was risky as it harbored higher levels of many glycolytic genes, higher age, IDH-wildtype rates, and higher rates of mesenchymal subtypes. To reduce model variable numbers, the glycolytic genes were filtered by univariate analysis. As a result, 11 genes (*GPI, LDHA, ENO1, PFKFB4, STAT3, FBP1, HK3, GCKR, HK1, ALDHA, JMJD8*) were screened out, and their levels were remarkably higher in cluster 1 as calculated in both CGGA-array (*n* = 124) and CGGA-seq (*n* = 108) datasets (Fig. 1F, G). These results demonstrated that the glycolytic genes were highly prognosis-related.

The mutual correlations of the 11 genes were quantified and visualized in Fig. 1H. Subsequently, the 11 glycolytic genes were further filtered by ENet regression, 7 genes (*LDHA, JMJD8, HK3, GCKR, FBP1, ENO1, ALDOA*) were reserved according to the lambda.min and their coefficients were obtained (Fig. 1I, J). The seven genes were all risky genes, with *ENO1* achieving the highest coefficient value, followed by *GCKR* and *LDHA*. GS = 0.147891545 × expression of ENO1 + 0.040414563 × expression of FBP1 + 0.005340805 × expression of ALDOA + 0.125568431 × expression of GCKR + 0.013597690 × expression of HK3 + 0.059675588 × expression of JMJD8 + 0.113565853 × expression of LDHA.

### High GS predicted poor survival and risky features of GBM patients

**Fig. 2:**
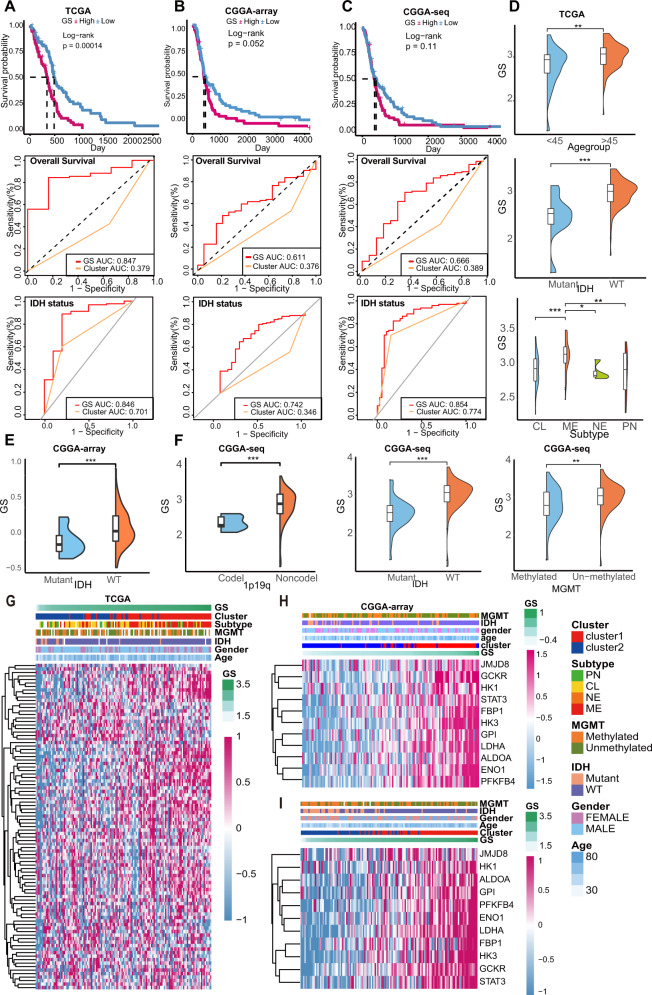
Estimation of the GS predictive capability for survival and risky features. **A**–**C** The GS predicted the overall survival of patients presented by the Kaplan–Meier curves and the discrimination estimating of OS, IDH prediction in TCGA (**A**), CGGA-array (**B**), and CGGA-seq (**C**)**. D** The GS differences between age > 45/≤45, IDH mutant/wildtype, and GBM subtype groups in the TCGA dataset. **E** The GS differences between IDH mutant/wildtype groups in CGGA-array dataset. **F** The GS differences between 1p/19q co-deletion/non-codeletion, IDH mutant/wildtype, MGMT methylated/unmethylated groups in CGGA-seq dataset. **G** Heatmaps of the glycolysis genes in TCGA patients ranked by their GS. **H**, **I** The 11 survival-related gene expressions in CGGA-array (**H**) and CGGA-seq datasets (**I**) ranked by their GS.

To test the predictive ability of the GS, survival analyses were performed. As exhibited in Fig. 2, the high-GS group patients harbored significantly lower survival rates than low-GS group patients, the GS showed a higher area under the curve (AUC) than clusters when predicting OS and IDH status in TCGA and CGGA-array datasets. Though without statistical significance, high-GS predicted shorted survival rates in the CGGA-array and the CGGA-seq dataset, and it predicted IDH status with higher discrimination (Fig. 2B, C). While the AUC of MGMT status was relatively low (Fig. S1A–S1C). Besides, the GS was higher in high age group, IDH wildtype, mesenchymal subtype patients in TCGA (Fig. 2D), IDH wildtype patients in CGGA-array (Fig. 2E), and IDH wildtype, 1p/19q non-codeleted, MGMT unmethylated patients in CGGA-seq dataset (Fig. 2F). The comparison of the AUC, C-index, and time-sequencing C-index showed that the performance of the GS outperformed the that of other two published glycolysis-related models for predictive glioblastoma patient OS. (Fig. S1D–S1F).

The glycolysis gene levels were then compared in TCGA patients ranged on their GS levels, many genes were upregulated as the GS increased (Fig. 2G). Particularly, the 11 survival-related genes expression increased with the GS in CGGA-array and CCGA-seq datasets (Fig. 2H, I). Notably, we performed SNV analyses and noticed that the high-GS group was associated with a higher SNV rate of the tumor suppressor gene *PTEN* (Fig. S2A–S2C). Since hypoxia can trigger the aberrate glycol-metabolism [38], we, therefore, investigated the hypoxia signature collected from literature and observed higher hypoxia levels as most of the hypoxia genes were upregulated in the high-GS group (Fig. S2D–S2F).

Taken together, the results demonstrated that the GS had a good capability in predicting survival, GBM clinical and molecular features, PTEN mutation, and hypoxia status.

### Functional analysis and immune infiltration landscape in GBMs

**Fig. 3:**
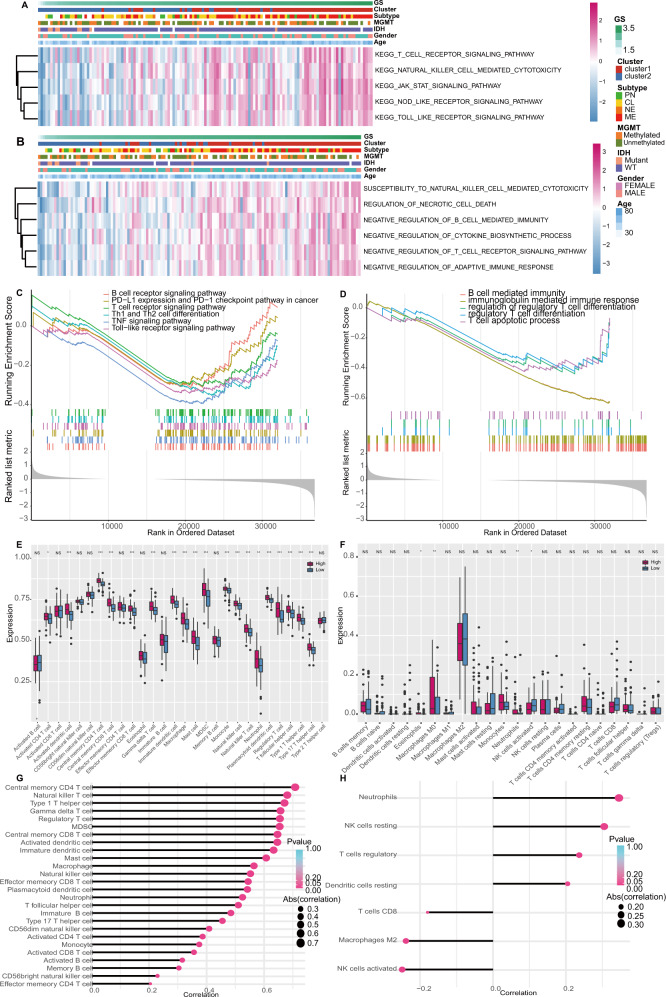
Functional analysis and immune infiltration estimation in TCGA dataset. **A**, **B** GSVA heatmap of the KEGG pathways (**A**) and GO biological processes (**B**) signatures stratified by patient features. **C**, **D** GSEA of the KEGG pathways (**C**) and GO processes (**D**). **E**, **F** Calculation of 28 TILs (**E**) and LM22 (**F**) infiltration differences between the high-GS and the low-GS group, the box plots plotted the median, first and third quartiles, minimum and maximum values. **G** and **H** Quantified correlation between the GS and 28 TILs infiltration (**G**) and between the GS and infiltration of immunocytes from LM22 (**H**).

To explore the differentially enriched activities in GBMs, we performed GSVA analysis to identify the highly variated pathways in KEGG and the biological processes from GO. The heatmap showed the involved pathways or biological processes ordered by GS. In KEGG, JAK-STAT, NOD-like receptor, and Toll-like receptor signaling pathways et al. were associated with higher GS, higher rates of cluster 1, mesenchymal subtype, IDH-wildtype, and higher age (Fig. 3A). In GO, negative regulation of cytokine biosynthetic process, negative regulation of B cell-mediated cytotoxicity, and negative regulation of T cell receptor signaling pathway et al. were observed to be associated with high GS, high rates of cluster 1, mesenchymal subtype, IDH-wildtype, and high age (Fig. 3B), suggesting the association between anti-cancer immune activity and the high-GS GBMs.

Further, the quantified enrichment scores of KEGG pathways, and GO biological processes were analyzed by GSEA. In KEGG, B cell, T cell, Th1/Th2 cell differentiation-related pathway et al. in cancer were downregulated (Fig. 3C). In GO, B cell-mediated immunity and immunoglobulin-mediated immune response et al. were also downregulated (Fig. 3D). The above functional analyses suggested that B cells and T cells-related anti-cancer immunity were affected in GBM with higher glycolysis level.

We further calculated an Enrichment Score of 28 TILs to discover the immune infiltration variation between the high-GS and the low-GS groups. As a result, many TILs were significantly infiltrated in the high-GS group compared to the low-GS group, such as central memory CD4 and central memory CD8 T cells, macrophages, MDSC, and Tregs et al. (Fig. 3E). The infiltration of 22 hematopoietic cell phenotypes by Cibersort calculation of LM22 was also presented. Three cell phenotypes were highly infiltrated in the high-GS group, including eosinophils, M0 macrophages, and neutrophils, while activated NK cell was lowly infiltrated (Fig. 3F). The quantified correlation between GS and 28 TILs and LM22 infiltration was visualized by a lollipop chart. In 28 TILs (Fig. 3G), the top 5 cells (central memory CD4 T cell, natural killer T cell, type 1 helper cell, gamma delta T cell, and regulatory T cell) were positively correlated with GS, which are engaged in T cell-mediated immunity and immune regulation. Particularly, Tregs may contribute to immune suppression. In the LM22 signatures, Tregs were positively correlated with GS, while CD8 T cells and activated NK cells were negatively correlated (Fig. 3H). The immunocyte infiltrating analyses demonstrated the suppression of T cells. Additionally, NK cells and Tregs were also detected and associated with immune suppression, manifesting a complex non-neoplastic network in the microenvironment. When immunocyte infiltration was validated in CGGA datasets, similar trends were observed (Fig. S3).

### Microenvironment scoring and immune escape gene expressions

**Fig. 4:**
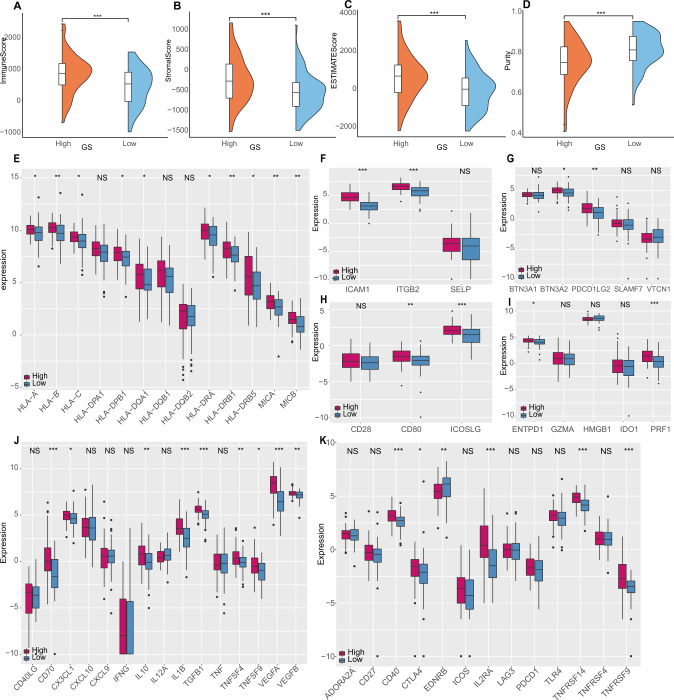
ESTIMATE Score calculation and immune escape gene expression. **A**–**D** The immune score (**A**), stromal score (**B**), combined ESTIMATE score (**C**), and the purity (**D**) calculation of the high-GS and the low-GS groups in the TCGA dataset. **E**–**K**: Box plot of immune escape gene expression differences between the low-GS (blue) and the high-GS (red) groups in TCGA dataset patients, including antigen presentation (**E**), adhesion (**F**), co-inhibition (**G**), co-stimulation (**H**), others **(I**), ligand (**J**), and receptor (**K**) related genes.

Since the previous immune pathways, biological processes, and immunocyte scans suggested a complex immune landscape and immune diversity between the high-GS and the low-GS groups, the whole cancerous microenvironment infiltrating levels deserved estimating. Apart from immune cells, stromal cells are also indispensable members of the tumor microenvironment, they comprise the essential non-tumor component in malignant tumor tissues. We thereby calculated a Stromal score and an Immune score via Estimation of Stromal and Immune cells in Malignant Tumor tissues using the Expression data (ESTIMATE) algorithm; the Immune score, Stromal score, and ESTIMATE score were higher in the high-GS group, as shown in Fig. 4A–C. Besides, the tumor purity exhibits the opposite distribution, which is lower in the high-GS group (Fig. 4D). In CGGA-seq and CGGA-array datasets, the same comparing results were also obtained (Fig. S4A–S4H). Hence, the high-GS GBMs were certified to bear a plethora of non-neoplastic cells.

The potential roles played by various non-tumor cells in GBMs may suppress anti-cancer immunity and escape tumor cells from elimination. Therefore, we detected the expression level of genes associated with immune escape [34]. The gene sets were associated with cell adhesion, antigen recognition, immune co-inhibition, co-stimulation, ligand, receptor, and others. As presented in box plots, the expression level of genes, including cell adhesion (*ICAM1*, *ITGB2*), antigen recognition (eight *HLAs, MICA*, and *MICB*), immune co-stimulation (*CD80, ICOSLG*), immune co-inhibition (*BTN3A2, PDCD1LG2*), others (*ENTPD1, PRF1*) were significantly higher in the high-GS group (Fig. 4E-4i). As for receptor and ligand genes, the expression level of 14 genes was significantly higher in the high-GS group, including 9 ligand-related genes (*CD70*, *CX3CL1*, *IL10*, *IL1B*, *TGFB1*, *TNFSF4*, *TNFSF9*, *VEGFA*, *VEGFB*) and 5 receptor-related genes (*CD40*, *CTLA4*, *IL2RA*, *TNFRSF14*, *TNFRSF9*) (Fig. 4J, K). The various immune escape gene up-expressions supported the emergence of immune-suppressive signatures in high-GS group GBMs.

### Functional validation of GS in single-cell RNA-seq dataset

**Fig. 5:**
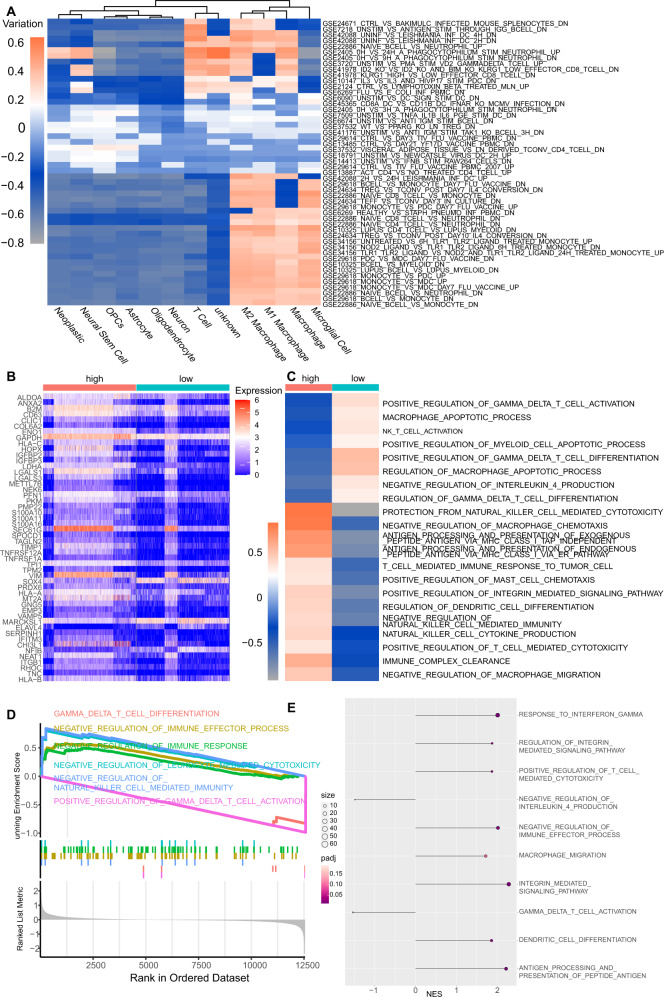
Functional analyses in single-cell datasets. **A** GSVA analysis of immune signatures for all clusters. **B** Heatmap of top 50 differentially expression genes divided by the high-GS and the low-GS in neoplastic cells. **C** GSVA analysis of GO biological process differences between the high-GS and the low-GS neoplastic cells. **D** GSEA of GO immune biological processes. **E** Lollipop plots depicting normalized enrichment scores of GO biological processes in the high-GS neoplastic cells.

The scRNA-seq datasets were integrated and analyzed. We ran a u-map reduction and eventually identified the cellular clusters based on markers and annotated the neoplastic cells by the Copykat results shown in Fig. S5. The GS and its seven glycolytic gene expressions were relatively highly expressed in neoplastic cells. To investigate the functional role of the GS in single cells, we first applied GSVA of immune signatures for all clusters; a similar immune signature pattern between neoplastic cells and neural stem cells, as well as a similar pattern among macrophages, was observed (Fig. 5A). Notably, the neoplastic cells, neural stem cells, T cells, and macrophages shared the same gene set variation, which was correlated to the escape of the neutrophil-mediated immunity by Anaplasma phagocytophilum [39]. This might suggest their correlation with immunity dysfunction. Next, we extracted neoplastic cells and divided them into the high-GS and the low-GS groups according to their median GS value. The top 50 DEGs exhibited apparent diversity as exhibited in the heatmap (Fig. 5B). The GSVA results of GO pathways enrichment also variated between the high-GS and the low-GS groups. In the high-GS group, the enrichment of positive regulation of T cell-related activation and differentiation, macrophage apoptosis et al. were lower, while some immune suppression associated biological processes were higher, for instance, negative regulation of macrophage chemotaxis and negative regulation of natural killer cell-mediated immunity et al. were highly enriched (Fig. 5C). The GSVA results preliminarily suggested a depressed immune situation involving T cell, natural killer cell, and macrophages in the high-GS neoplastic cells. We then conducted GSEA for seeking the enrichment levels of GO immune biological processes in the high-GS group. GSEA plots showed that gamma delta T cell differentiation and activation were downregulated and negative regulation of NK cells activity, immune effector process, immune response, and cytotoxicity were highly enriched (Fig. 5D). The lollipop plot exhibited differentially enriched immune processes with their normalized enrichment scores, we noticed that gamma delta T cell activation signaling pathways were negatively enriched in the high-GS group, and negative regulation of immune effector process and macrophage migration signaling pathways were highly enriched (Fig. 5E). The similar results obtained from GSEA and GSVA manifested that the high-GS neoplastic cells may be associated with immunosuppression involving macrophage, natural killer cell, and T cells.

### The high-GS neoplastic cells interacted with macrophages and microglia

**Fig. 6:**
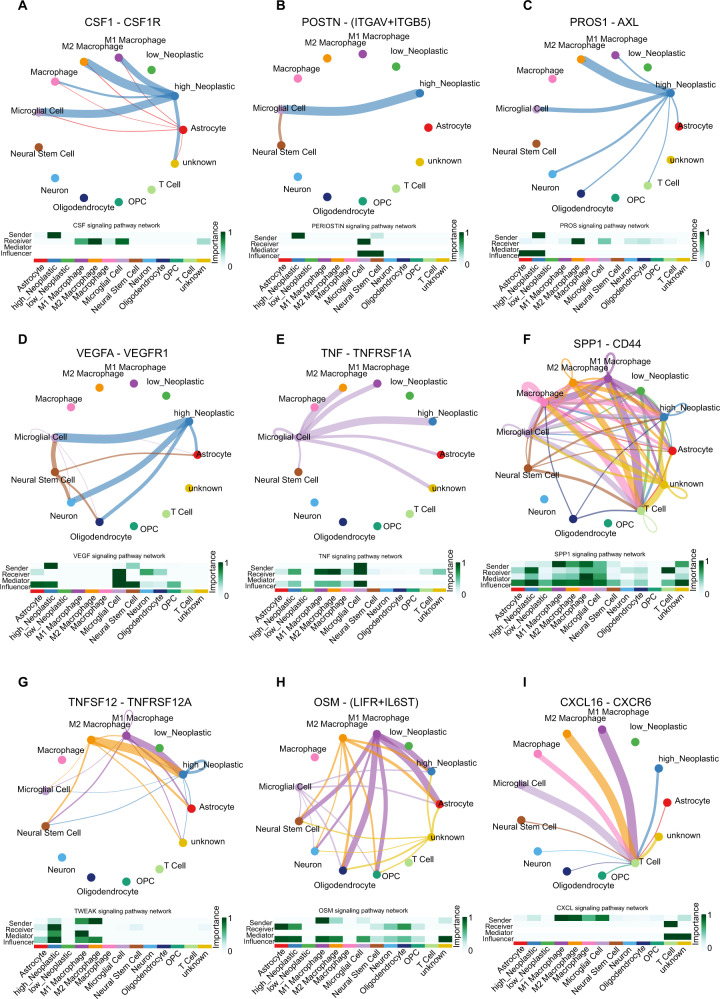
Intercellular cross-talk of all single-cell clusters. Cross-talk circus plot depicts the intercellular interaction net (upper), and the heatmap shows the role importance of each cluster in the signaling pathways mediated by CSF1/CSF1R (**A**), POSTN/(ITGAV and ITGB5) (**B**), PROS1/AXL (**C**),VEGFA/VEGFR1 (**D**), TNF/TNFRSF1A (**E**), SPP1/CD44 (**F**),TNFSF12/TNFRSF12A (**G**), OSM/(LIFR and IL6ST) (**H**), and CXCL16/CXCR6 (**I**).

Previous steps suggested the diverse immunological status between the high-GS and the low-GS groups. We then conducted CellChat analysis to directly seek the cross-talk among all cellular clusters based on ligand-receptor pairs. The global outcoming and incoming communication patterns of secreting and target cells were shown in Fig. S6A, S6B. The circle plots and cellular role heatmaps depicting intercellular cross-talk showed the interaction between macrophages, microglia, and high-GS neoplastic cells (Fig. 6A–H). The high-GS neoplastic cells were observed to affect macrophages and microglia via many signaling pathways as senders, who secreted the ligands that bind to the corresponding receptors of receivers. The high-GS neoplastic cells targeted microglia in CSF1/CSF1R, POSTIN/ (ITGAV and ITGB5), PROS1/AXL, and VEGFA/VEGFR1 mediated signaling pathways (Fig. 6A–D), and affected M2 macrophages in CSF1/CSF1R and PROS1/AX1 mediated signaling pathways (Fig. 6A, C). Correspondingly, microglia and M2 macrophages can also modulate the high-GS neoplastic cells. Microglia modulated the high-GS neoplastic cells in TNF/TNFRSF1A and SPP1/CD44 mediated signaling pathways (Fig. 6E, F). For M2 macrophage, they modulated the high-GS neoplastic cells in SPP1/CD44, TNFSF12/TNFRSF12A, and OSM/ (LIFR and IL6ST) mediated signaling pathway (Fig. 6F–H). Additionally, we also noticed that the high-GS neoplastic cells, microglia, and macrophages affected T cells in CXCL16/CXCR6 mediated pathway (Fig. 6I). Shortly, these results manifested that the high-GS neoplastic cells harbor abundant cross-talks with microglia, M2 macrophages, and T cells.

### ENO1 modulated glioblastoma’s effects on microglia M2 polarization via co-culture

**Fig. 7:**
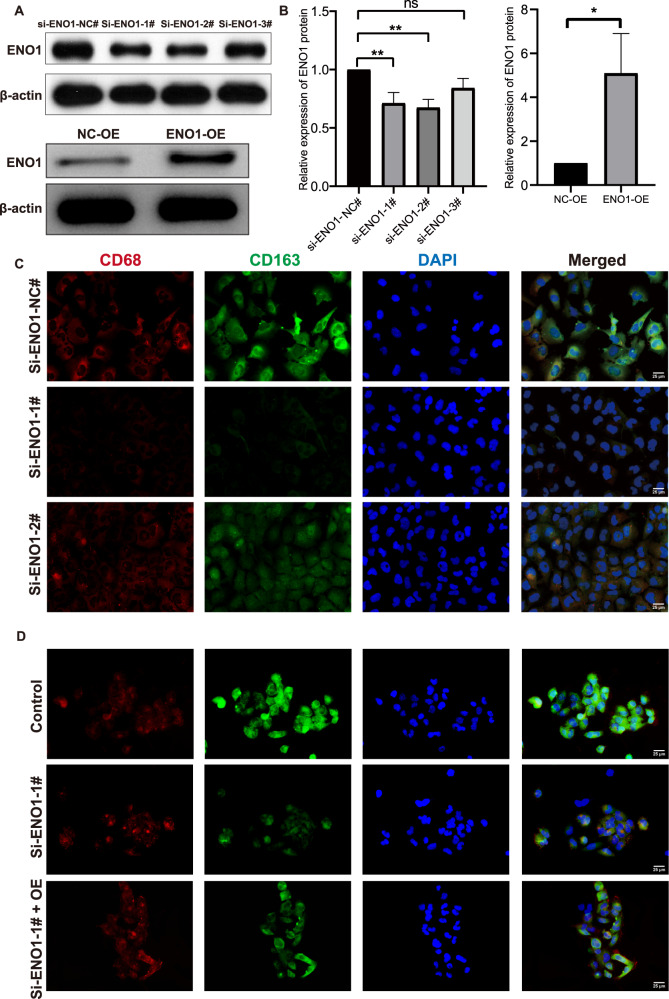
Glioblastoma ENO1 affected microglia M2 polarization. **A** The western-blot bands of ENO1 and β-actin in si-ENO1-NC#, si-ENO1-1#, si-ENO1-2#, si-ENO1-3# groups (upper) and bands of ENO1 and β-actin in NC-OE and ENO1-OE groups (lower). **B** The relative expression of ENO1 protein by a statistical summary of three times of independent western-blot experiments. **C**, **D** Multiple fluorescence staining of the nucleus (DAPI blue), CD68 (red), CD163 (green) on M2 macrophages co-cultured with ENO1-knockdown/siRNA-control (**C**) and ENO1 siRNA/overexpression rescued T98G cells (**D**). The Merged (the rightmost panel) refers to the merged image of CD68, CD163, and DAPI panels.

To validate that glycolysis was involved in the cross-talk between glioblastoma and microglia, glioblastoma cell ENO1-knockdown and ENO1 overexpression co-culture with microglia were performed. The ENO1 protein expression was significantly decreased in si-ENO1-1#, si-ENO1-2#, and increased in ENO1-OE T98G cells detected by western blotting (Fig. 7A, B), and the immunofluorescence results presented that the CD163 fluorescence intensity was decreased in both si-ENO1-1#, si-ENO1-2# T98G groups (Fig. 7C). The following rescue experiment exhibited that overexpression of ENO1 can ease the CD163 downregulation caused by ENO1-knockdown (Fig. 7D). These demonstrated that ENO1 in glioblastomas contributed to macrophage M2 polarization.

### ENO1 promoted glioblastoma cell clone formation, cell viability, and migration

**Fig. 8:**
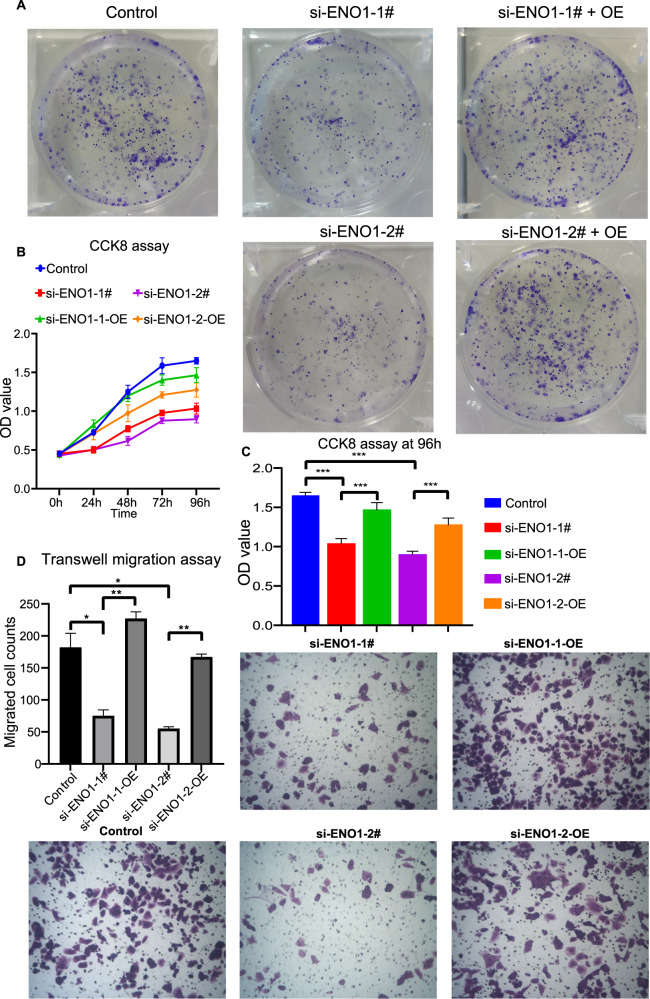
Glioblastoma ENO1 affected cancer cell malignant behaviors. **A** The images of clone formation count. **B** The line chart exhibits the optical density change at different time points in CCK8 assays. **C** The comparison of the optical density value among all groups at 96 h. **D** The images of the crystal violet stained cells that migrated through the transwell membrane and the quantified comparison of migrated cells.

To investigate whether ENO1 affects the cancer cell malignant behaviors, we also performed function rescue experiments using T98G cells. The clone formation assay images depicted a trend that the clone formation was depressed in both ENO1-knockdown groups, and ENO1 overexpression rescued this depression (Fig. 8A). Similarly, the CCK8 assay results exhibited the ENO1-OE-rescued cell viability in both two siRNA-knockdown groups at 96 h (Fig. 8B, C). For cell migration capability, we also observed that ENO1 overexpression reversed the depressed cell migration ability caused by ENO1-knockdown in both two groups (Fig. 8D). The function rescue results confirmed that ENO1 was engaged in promoting glioblastoma growth and migration.

## Discussion

Malignant gliomas confer a growth advantage via the regulation of glycolysis. In this study, We identified 11 genes by univariate analysis, and the 11 genes were further reduced to 7 genes by Elastic Net regression. The seven genes were then used to build the GS for prognosis prediction. Previously, Chen et al. [12] and Wang et al. [13] published glycolytic signatures to predict GBM prognosis. However, their performance was not estimated. Discrimination verification is essential for model estimation [40]; here, our AUC values of predictive subjects manifested good accuracy, demonstrating that our model was presented with higher quality compared according to the criteria we previously proposed [41]. Also, our model performance outperformed that of Chen’s and Wang’s models according to the results of AUC and C-index comparisons, manifesting the good prognostic value of the GS.

We identified 7 risky glycolytic genes that were firmly and independently associated with survival. Among them, *GCKR* has not been reported as a prognostic biomarker so far; only two studies have reported that gastric and colorectal cancer patients with *GCKR* variates harbored a better prognosis than those of wildtype [42, 43], and our study revealed its prognostic value in gliomas for the first time*. ENO1* encodes α-enolase functioning in glycolytic processes; its knockdown reversed the malignant growth, and migration of glioma cells to normal levels [44]. Here, we further confirmed *ENO1’s* contributions to glioblastoma malignant behaviors via a series of rescue experiments, manifesting its critical role in glioblastoma progression. Also, we previously discovered *JMJD8* as a risk factor for many cancers, including glioma [45], and it is consistent with the discoveries in this study. For the other 4 genes, *FPB1* is a rate-limiting enzyme of gluconeogenesis, it was highly expressed in cancers and positively correlated with c-myc to facilitate glioma cell growth [46]. *LDHA* produces lactate during aerobic glycolysis and enhanced GBM cell variability [47], and *ALDOA* catalyzes fructose-1,6-bisphosphate into dihydroxyacetone phosphate and glyceraldehyde-3-phosphate and promotes GBM invasion [48]. The prognostic roles of these three genes identified in our study endorsed the previous findings [12, 46, 47, 49, 50]. As for *HK3*, its role in cell viability was not significant [51], but more investigations are deserved considering its emerging roles in other cancers [52, 53].

The interactions of glycolytic signatures with other biological processes in GBMs should be revealed for developing novel therapeutic strategies. Here, we noticed the high hypoxia status in the high-GS group. Hypoxia was associated with aerobic glycolysis, and it is also associated with GBM immunology [38]. Previously, Zhou et al. [14] enriched a series of immunological functions through an energy metabolism genes signature. However, no further analyses were conducted, letting the association between immune and glycolysis to remain unclear. In our study, we found the association between anti-cancer immunity and the high-GS group in the gene set, cellular, and molecular levels; the bulk sequencing results showed the negative regulation of T cell and B cell-related activation in the high-GS group. The higher Tregs and lower CD8 + T cells, NK cell infiltration in the high-GS group, suggested the immunosuppressive status mediated by Tregs [54]. In single-cell datasets, we also found that NK cells, normal macrophage chemotaxis, and T cell differentiation and activation and were suppressed in the high-GS GBMs. Taken together, the high glycolysis level seemed to produce extensive immune destruction induced by rather neoplastic cells or immune-suppressive cells, which may be responsible for the malignant phenotypes and prognosis in the high-GS group patients.

The CellChat results further support the interplays between the high-GS neoplastic cells and non-neoplastic cells, which mainly lay in several immune-related signaling pathways. Various studies have been reported to support glioblastoma’s effects on macrophages/microglia. Macrophages rely on CSF1–CSF1R for survival and differentiation, and CSF1R inhibition suppressed glioblastoma progression both in vivo and in vitro, and, importantly decreased the macrophage M2 marker expression [55]. Glioblastoma-secreted periostin encoded by POSTN recruited M2 macrophages to promote cancer growth [56]. Similarly, inhibiting highly expressed VEGFR1 on glioma-associated macrophages/microglia reduced glioblastoma angiogenesis and prolonged the survival of tumor-bearing mice [57], and high expression of SPP1-CD44 promoted macrophage infiltration, leading to poor prognosis [58]. Reversely, macrophages/microglia also modulate glioblastoma cells. Macrophage-derived OSM promoted glioblastoma cells' mesenchymal transition by targeting LIFR [59]. This evidence presented great accord with our discoveries in the CellChat results. And for PROS, it was secreted by tumor-associated macrophages and targeted AXL on glioma stem cells, while we discovered that the high-GS also functioned as the sender to interact with M2 macrophages [60], suggesting the PROS-AXL-mediated mutual interplay between macrophage and glioblastoma cells. Since no study of TNF-TNFRSF1A and TNFSF12-TNFRSF12A has been reported so far, our study discovered novel glioblastoma-macrophage/microglia interactive patterns. Altogether, the ligand–receptor-based interaction found in this study and from the literature strongly demonstrates the cross-talk between the high-GS glioblastoma cells and M2 macrophage/microglia, and the glioblastoma-derived *ENO1*’s roles in regulating microglia M2 polarization was subsequently verified by rescue experiment here, making their exposed interaction a promising target for further therapeutic strategy development. Interestingly, we also noticed that the high-GS neoplastic cells and macrophages/microglia targeted T cells via CXCL16-CXCR6. This pair induced vascular epithelial cell-mediated T cell adhesion and was also expressed on microglia and glioma cells to promote microglia proliferation, whereas its roles in the cross-talk between macrophages/microglia, the high-GS neoplastic cells, and T cells remained unknown, which requires further exploration [61].

At the genetic level, we compared the immune escape gene expression between the two GS groups, and concordant results of higher expression levels in the high GS group of most immune escape genes were found, highlighting the immunosuppressive progress in the GBMs environment.

Finally, we discuss the single gene’s immune effect in our GS, aiming at clarifying the immune effects of the critical genes in glycolysis. *LDHA* promoted lactate acid acceleration to form an immunosuppressive environment, depressing inflammation via its effects on T cells, macrophages, and natural killer cells in various cancers [62–65]. Accelerated lactic acid enables tumors to skew macrophages towards M2 phenotypes [66, 67]. *HK3* was associated with the inflammatory response in non-small cell lung cancer and predicted anti-PD1 immunotherapy [52]. Aberrant *FBP1* expression weakened the anti-cancer progression of natural killer cells by destroying their normal metabolic capability and impairing viability [68], while its inhibition can restore natural killer cell function. For *JMJD8*, we recently discovered its potential as an M2 macrophage marker [45]. Little experimental evidence was reported about *ALDOA* ‘s association with immunocytes in cancers, but its potential roles in B cells, CD4+ T cells, and Th cells were noticed by GSEA [69]. A pan-cancer study showed that *ENO1* was negatively correlated with B cells and macrophages in cervical carcinoma and B cells in lung adenocarcinoma [70]. Its inhibitor also activated CD8 + T cells and natural killer cells in multiple myeloma [71]. But so far, no direct evidence has been presented for *ENO1*’s immunological roles. Herein, *ENO1* was identified as the key risky gene with the highest coefficient. Importantly, we confirmed that *ENO1* affected M2 microglia polarization for the first time. Thus, our study revealed the immunological roles of the GS genes in glioblastoma, and these genes will serve as promising targets of anti-cancer therapy.

In conclusion, we construct a robust GS and found that the high GS can predict malignant features and unfavorable survivals with high accuracy and repeatability. The bulk and single-cell analyses of the interaction between high glycolysis and the immune revealed a plethora of immunosuppressive activities mainly associated with T cells, B cells, natural killer cells, and macrophages/microglia, and we confirmed that ENO1 promoted microglia M2 polarization and glioblastoma cell progression. These jointly constructed a strong protective umbrella against anti-cancer supervision and resulted in the growth of neoplastic cells. Clinical application of the GS and the understanding of these cross-talks can improve prognostic management and provide us with novel targets for anti-cancer therapy, thus making GBM patients beneficial.

## Supplementary information

Figure S1

Figure S2

Figure S3

Figure S4

Figure S5

Figure S6

Supplementary Material S1

## Data Availability

The datasets used in this study can be obtained from TCGA; CGGA; GEO; Single Cell Portal. The R code can be retrieved from the corresponding authors.

## Code availability

The R codes used in this study can be retrieved from the corresponding authors.
