# Supplementary material for: Glioblastoma glycolytic signature predicts unfavorable prognosis, immunological heterogeneity, and ENO1 promotes microglia M2 polarization and cancer cell malignancy
Source: Cancer Gene Ther. 2022 Dec 9;30(3):481–96. doi: 10.1038/s41417-022-00569-9 (PMC10014583; doi:10.1038/s41417-022-00569-9)
Supplement: Supplementary file 1 — Figure S1 [file 41417_2022_569_MOESM1_ESM.pdf]

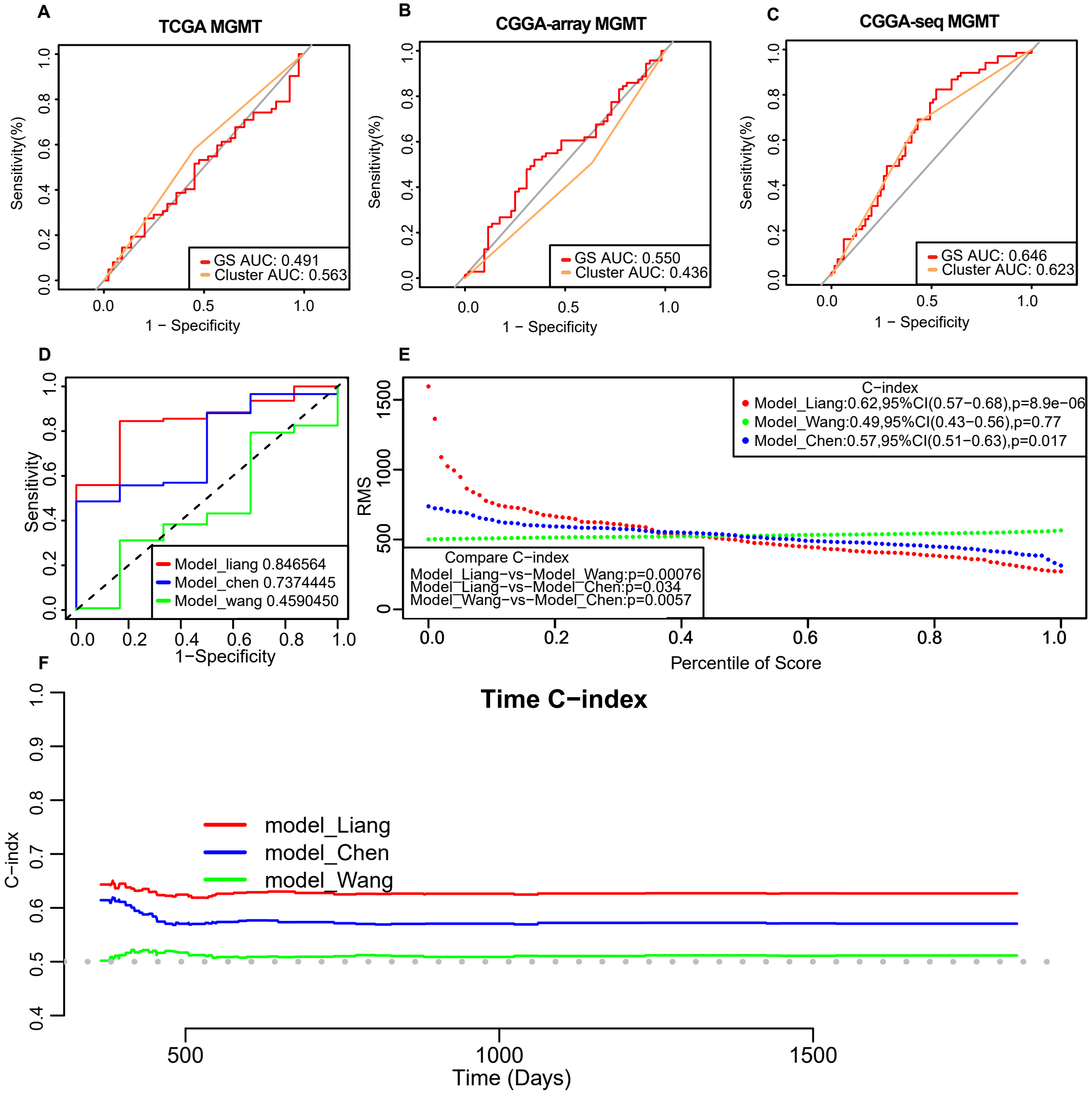

Figure S1: **Performance estimation and comparison of the Glycolytic Score.**

A-C: Receiver operating curves for MGMT methylation predictions by Glycolytic Score and PCA clusters in TCGA (A), CGGA-array (B), and CGGA-seq (C). D-F: Comparison of AUC (D), C-index (E), and time-sequencing C-index (F) for predicting glioblastoma patient OS among three established glycolysis-related prognostic model.
