# Supplementary material for: Glioblastoma glycolytic signature predicts unfavorable prognosis, immunological heterogeneity, and ENO1 promotes microglia M2 polarization and cancer cell malignancy
Source: Cancer Gene Ther. 2022 Dec 9;30(3):481–96. doi: 10.1038/s41417-022-00569-9 (PMC10014583; doi:10.1038/s41417-022-00569-9)
Supplement: Supplementary file 2 — Figure S2 [file 41417_2022_569_MOESM2_ESM.pdf]

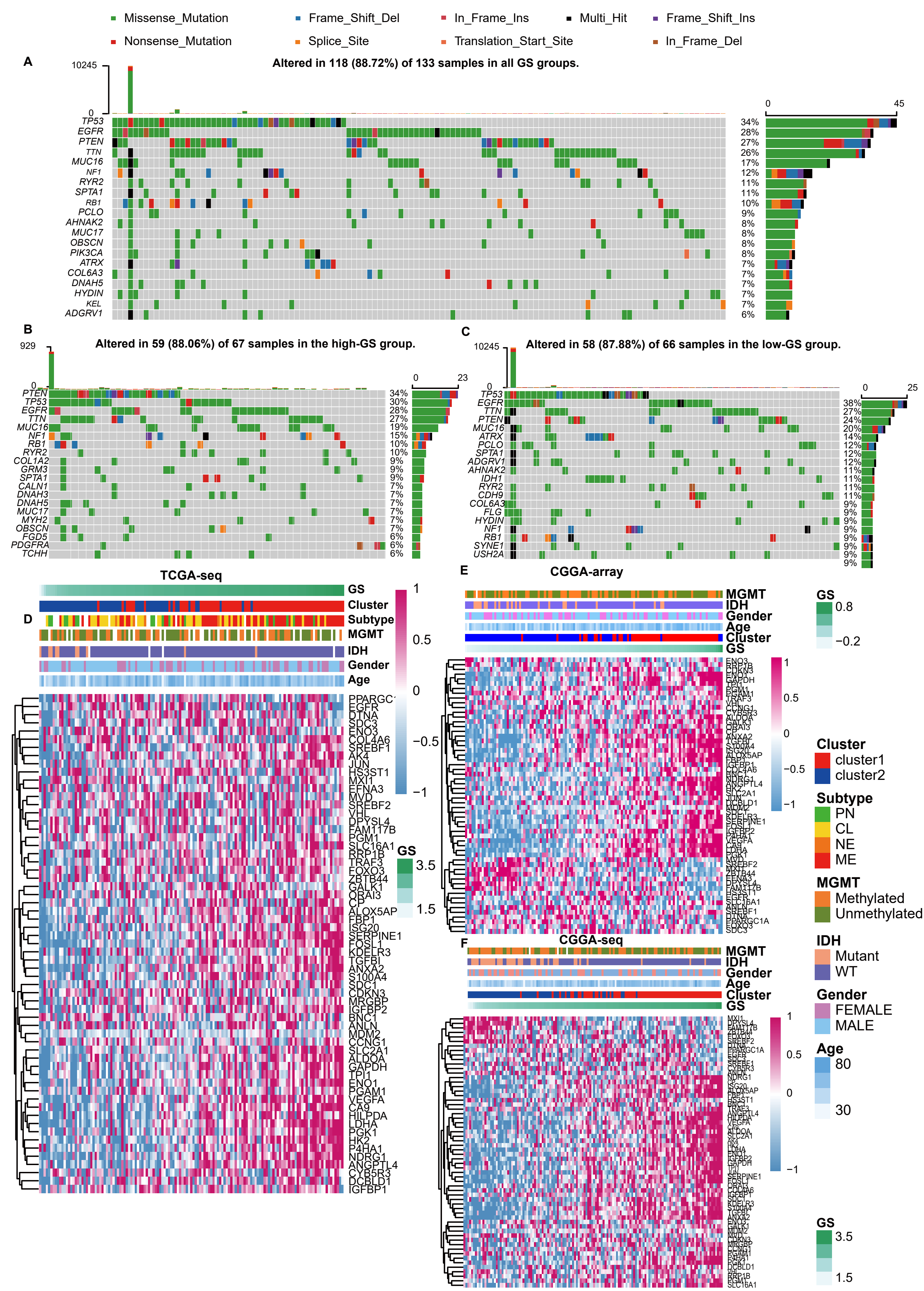

Figure S2: **Single nucleotide variant differences and hypoxia status separated by the Glycolytic Score groups.**

A: The top 20 genes with most single nucleotide polymorphisms of the whole TCGA dataset. B: The top 20 genes with most single nucleotide polymorphisms of TCGA dataset high-GS group. C: The top 20 genes with most single nucleotide polymorphisms of TCGA low-GS group, different mutations, and their c orresponding color labels were exhibited on the top. D-F: The hypoxia signature distribution ranged by GS in TCGA (D), CGGA-array (E), and CGGA-seq (F) datasets respectively. TCGA, The Cancer Genome Atlas; CGGA, Chinese Glioma Genome Atlas; GS, Glycolytic Score.
