# Supplementary material for: Glioblastoma glycolytic signature predicts unfavorable prognosis, immunological heterogeneity, and ENO1 promotes microglia M2 polarization and cancer cell malignancy
Source: Cancer Gene Ther. 2022 Dec 9;30(3):481–96. doi: 10.1038/s41417-022-00569-9 (PMC10014583; doi:10.1038/s41417-022-00569-9)
Supplement: Supplementary file 3 — Figure S3 [file 41417_2022_569_MOESM3_ESM.pdf]

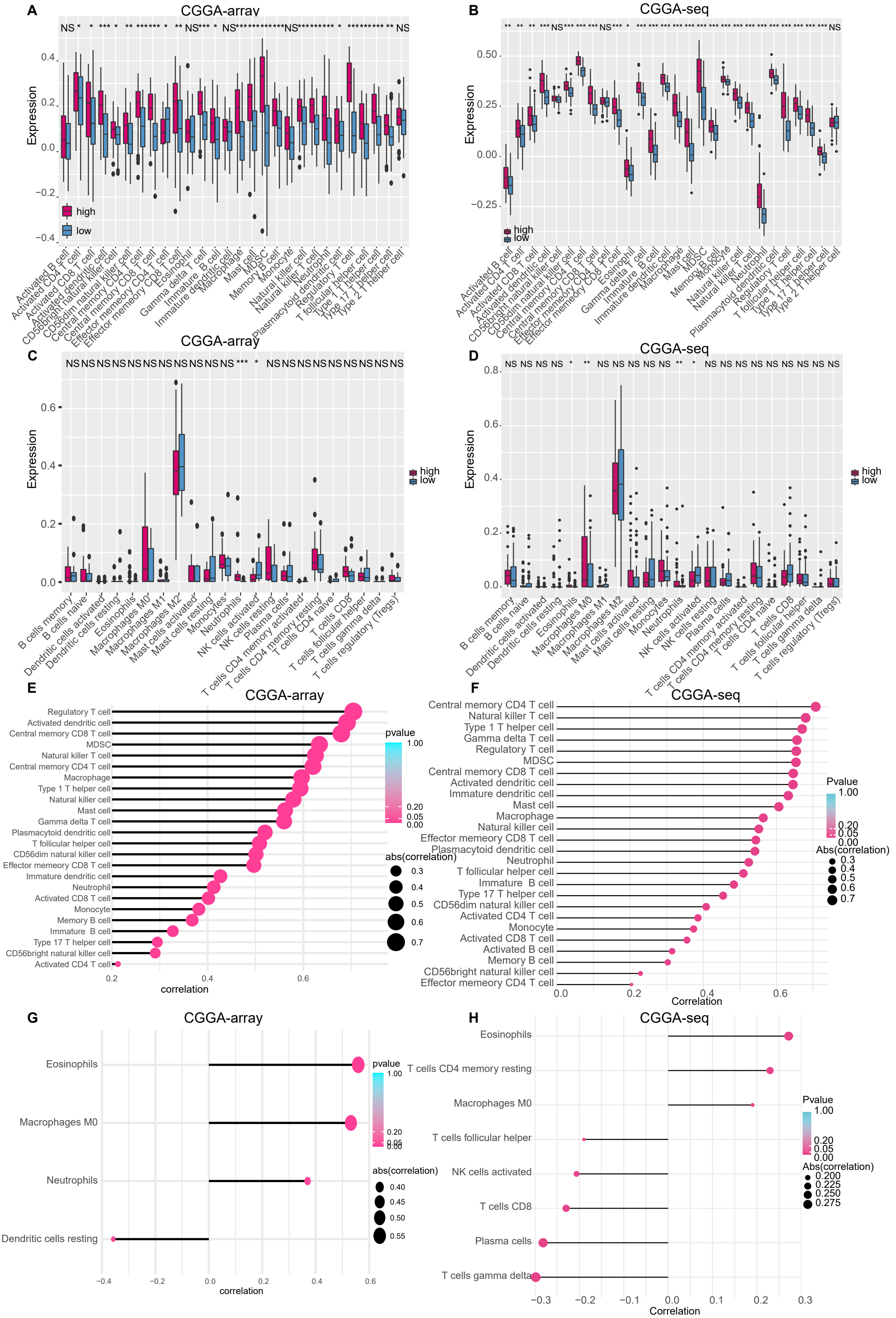

Figure S3: **Immunocytes infiltration analyses in validating datasets.**

A, C, E, G: 28 TILs (A), LM22 (C) calculation between high-GS and low-GS groups, the correlation between 28 TILs and GS (E), LM22 and GS (G) in CGGA-array dataset respectively. B, D, F, H: 28 TILs (B), LM22 (D) calculation, correlation between 28 TILs and GS (F), LM22 and GS (H) in CGGA-seq dataset respectively. CGGA, Chinese Glioma Genome Atlas; Abs, absolute; GS, Glycolytic Score.
