# Supplementary material for: Glioblastoma glycolytic signature predicts unfavorable prognosis, immunological heterogeneity, and ENO1 promotes microglia M2 polarization and cancer cell malignancy
Source: Cancer Gene Ther. 2022 Dec 9;30(3):481–96. doi: 10.1038/s41417-022-00569-9 (PMC10014583; doi:10.1038/s41417-022-00569-9)
Supplement: Supplementary file 4 — Figure S4 [file 41417_2022_569_MOESM4_ESM.pdf]

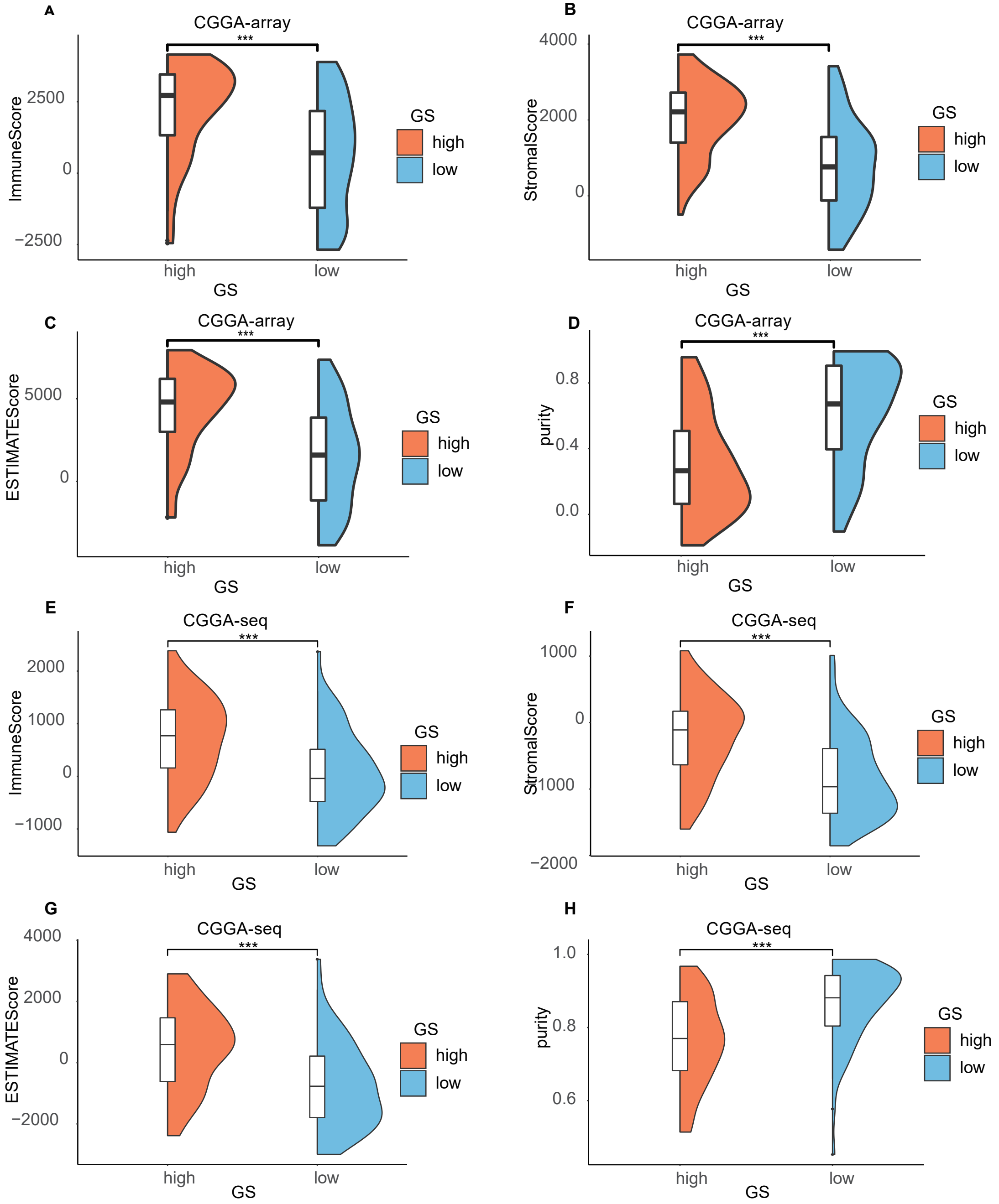

Figure S4: **ESTIMATE differences between high-GS and low-GS groups in validating datasets.**

A-D: The differences of Immune Score (A), Stromal Score (B), ESTIMATE Score (C), and tumor purity (D) between high-GS and low-GS groups in CGGA-array dataset. E-H: The differences of Immune Score (E), Stromal Score (F), ESTIMATE Score (G), and tumor purity (H) between high-GS and low-GS groups in the CGGA-seq dataset. TCGA, The Cancer Genome Atlas; CGGA, Chinese Glioma Genome Atlas; ESTIMATE, estimation of stromal and immune cells in malignant tumor tissues using expression data; GS, Glycolytic Score.
