# Supplementary material for: Glioblastoma glycolytic signature predicts unfavorable prognosis, immunological heterogeneity, and ENO1 promotes microglia M2 polarization and cancer cell malignancy
Source: Cancer Gene Ther. 2022 Dec 9;30(3):481–96. doi: 10.1038/s41417-022-00569-9 (PMC10014583; doi:10.1038/s41417-022-00569-9)
Supplement: Supplementary file 5 — Figure S5 [file 41417_2022_569_MOESM5_ESM.pdf]

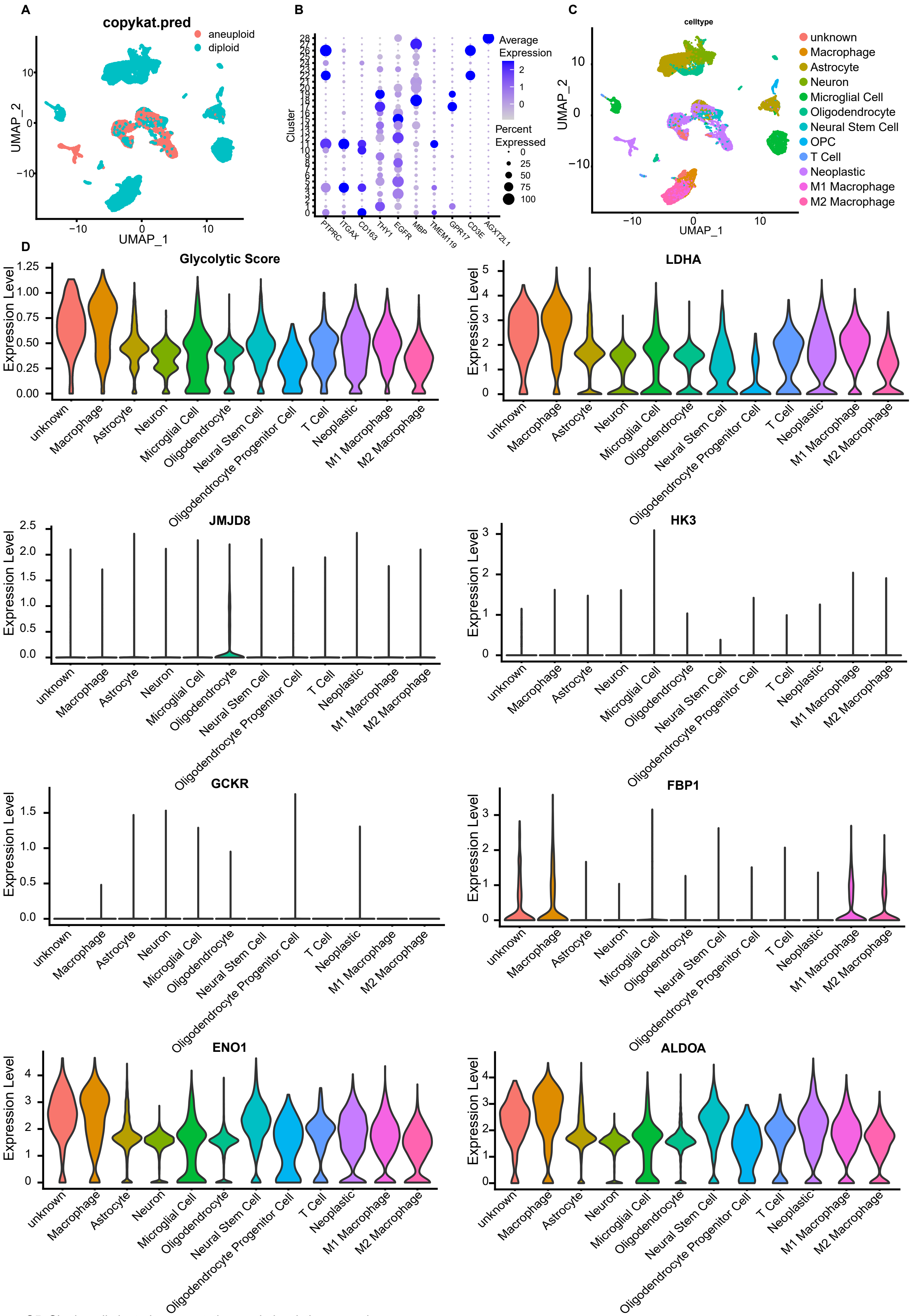

Figure S5: Single-cell clustering, annotation, and glycolytic expressions.

A: The Copykat results identifies cells with aneuploid feature. B: Dot plot of markers for all clusters annotations, size of the dots means the marker expression percentage of all cells in the corresponding cluster. C: The U-map reduction exhibits the cluster annotation for all cells. D: Presentation of the Glycolytic Score, LDHA, JMJD8, HK3, GCKR, FBP1, ENO1, ALDOA expressionof cells in each cluster.
