# Supplementary figures and images for: Glioblastoma glycolytic signature predicts unfavorable prognosis, immunological heterogeneity, and ENO1 promotes microglia M2 polarization and cancer cell malignancy
Source: Cancer Gene Ther. 2022 Dec 9;30(3):481–96. doi: 10.1038/s41417-022-00569-9 (PMC10014583; doi:10.1038/s41417-022-00569-9)

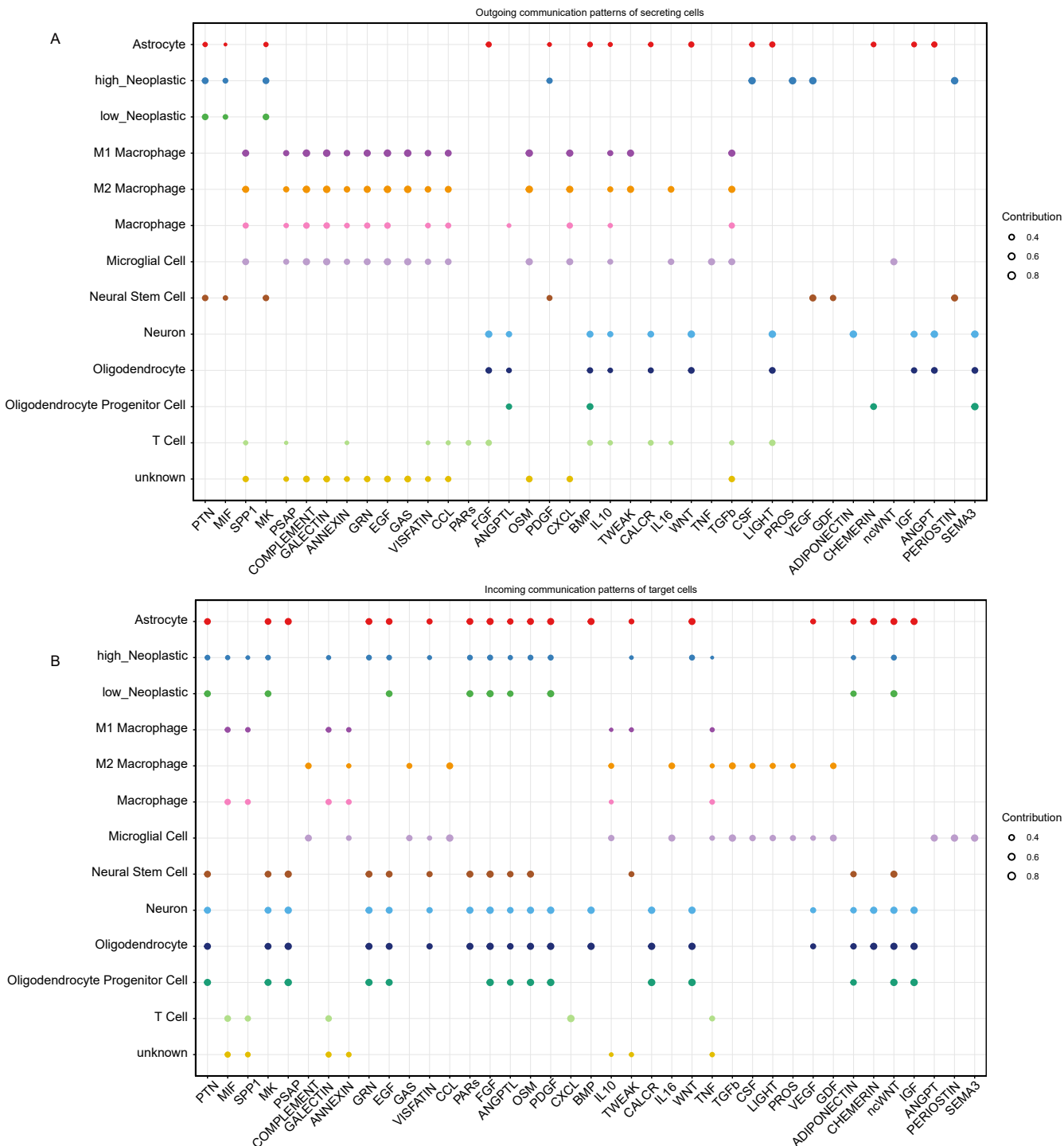

Supplement: Supplementary file 6 — Figure S6 [file 41417_2022_569_MOESM6_ESM.pdf]
