# Supplementary material for: Glioblastoma glycolytic signature predicts unfavorable prognosis, immunological heterogeneity, and ENO1 promotes microglia M2 polarization and cancer cell malignancy
Source: Cancer Gene Ther. 2022 Dec 9;30(3):481–96. doi: 10.1038/s41417-022-00569-9 (PMC10014583; doi:10.1038/s41417-022-00569-9)
Supplement: Supplementary file 7 — Supplementary Material S1 [file 41417_2022_569_MOESM7_ESM.pdf]

Supplementary Material S1. The siRNA sequences of *ENO1*.

T98G/si-ENO1-1#
Sense (5'-3'): CGUGAACGAGAAGUCCUGCAATT;
Antisense (5'-3'): UUGCAGGACUUCUCGUUCACGTT.

T98G/si-ENO1-2#
Sense (5'-3'): CCGGCGUUCAAUGUCAUCAAUTT;
Antisense (5'-3'): AUUGAUGACAUUGAACGCCGGTT.

T98G/si-ENO1-3#
Sense (5'-3'): CCACUGUUGAGGUUGAUCUCUTT;
Antisense (5'-3'): AGAGAUCAACCUCAACAGUGGTT.
